# Integrating Pressure-Driven Membrane Separation Processes to Improve Eco-Efficiency in Cheese Manufacture: A Preliminary Case Study

**DOI:** 10.3390/membranes10100287

**Published:** 2020-10-15

**Authors:** Scott Benoit, Julien Chamberland, Alain Doyen, Manuele Margni, Christian Bouchard, Yves Pouliot

**Affiliations:** 1 STELA Dairy Research Centre, Institute of Nutrition and Functional Foods, Department of Food Science, Université Laval, Québec, QC G1V 0A6, Canada; julien.chamberland@fsaa.ulaval.ca (J.C.); alain.doyen@fsaa.ulaval.ca (A.D.); Yves.Pouliot@fsaa.ulaval.ca (Y.P.); 2 International Reference Centre for the Life Cycle of Products, Processes and Services, Polytechnique Montréal, Department of Mathematical and Industrial Engineering, Montréal, QC H3C 3A7, Canada; manuele.margni@polymtl.ca; 3 Department of Civil Engineering and Water Engineering, Université Laval, Québec, QC G1V 0A6, Canada; Christian.Bouchard@gci.ulaval.ca

**Keywords:** eco-efficiency, process simulation, dairy processing, milk standardization

## Abstract

Pressure-driven membrane separation processes are commonly used in cheese milk standardization. Using ultrafiltration (UF) or microfiltration (MF), membrane separation processes make it possible to concentrate the milk proteins and increase the yields of cheese vats. However, the contribution of membrane separation processes to the environmental impact and economical profitability of dairy processes is still unclear. The objective of this study was to evaluate the contribution of membrane separation processes to the eco-efficiency of cheddar cheese production in Québec (Canada) using process simulation. Three scenarios were compared: two included UF or MF at the cheese milk standardization step, and one did not incorporate membrane separation processes. The results showed that even if membrane separation processes make it possible to increase vat yields, they do not improve the eco-efficiency of cheddar cheese processes. However, membrane separation processes may benefit the eco-efficiency of the process more when used for byproduct valorization.

## 1. Introduction

Pressure-driven membrane separation processes are widely used in the dairy industry. They allow for the concentration and purification of most dairy fluid components and enable the production of many high-value-added dairy products and ingredients [1,2,3]. Even though they improve manufacturing yields and produce high-priced ingredients, membrane separation processes water and energy and generates byproducts. Although the beneficial impact of their integration in many dairy processes on the economics is often put forward and even documented [4], it has not been demonstrated that these economics benefits are linked to reductions of the environmental impacts of the dairy processes. There are indeed no available studies on the implications of membrane separation processes on the environmental impacts and economic profitability of dairy processes. Environmental impact and economic profitability are the two dimensions taken into account in the eco-efficiency (EE) concept. It aims at delinking the creation of value from the use of natural resources and emission of pollutants [5], and carrying out EE assessments in compliance with ISO standards [6] makes it possible to assess simultaneously the economics and potential environmental impacts of a product or process. Accordingly, the broad aim of this paper was to provide some answers to the question of the contribution of the membrane separation processes to the eco-efficiency of the dairy processes.

In cheesemaking, membrane separation processes have been commonly used for cheese milk standardization for over 40 years [7]. Ultrafiltration (UF) is the most widely used technology since it increases the milk protein content (casein and whey proteins) prior to cheesemaking [8,9]. Standardization is usually carried out by adding UF retentate to unconcentrated milk [10]. Today, UF retentate is gradually being replaced by microfiltration (MF) retentate [11]. MF membranes of 0.1 µm pore size permit higher whey protein transmission while ensuring casein retention [12]. In addition, MF at the cheese milk standardization stage could improve the organoleptic qualities (taste and texture) of the cheese produced [11,13].

Therefore, the objective of this study was to evaluate, through a case study, the contribution of membrane separation processes to the EE of three scenarios of cheddar production in Québec (Canada). One scenario included UF at the cheese milk standardization stage, one used MF instead of UF at the cheese milk standardization stage, and one was devoid of any membrane separation processes. Cheddar manufacture was selected as a case study since it is the most produced cheese in Canada with 162,000 t produced in 2017—this was 33% of the Canadian cheese production. The Québec province is also the largest cheddar producer with an average of 45% of Canadian cheddar production between 2012 and 2017 [14]. The three scenarios were assessed by process simulation, using the EE assessment tool introduced by Benoit et al. [15]. It is based on a life-cycle assessment and was specifically developed for the dairy processing industry.

## 2. Materials and Methods

### 2.1. Processing Scenarios

**Figure 1:**
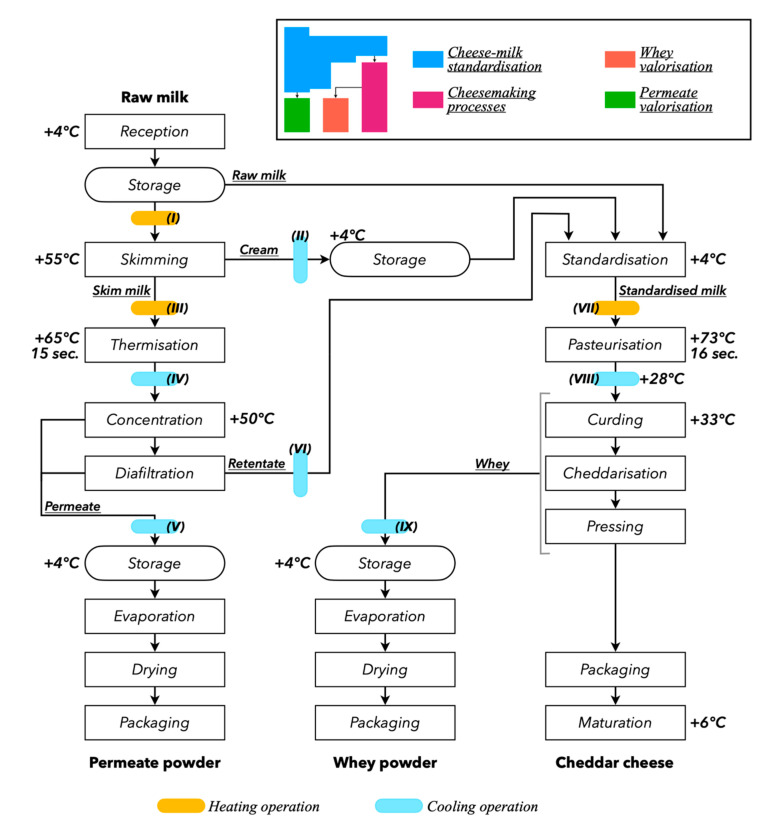
Process flow diagram of scenarios A and B. In scenario A, the concentration step is carried out using ultrafiltration membranes (10 kDa) until a volume concentration factor (VCF) of 3.5 is reached, followed by continuous diafiltration (2.0 DV). In scenario B, the concentration step is carried out using microfiltration membranes (0.1 µm) until a VCF of 3.0 is reached, followed by discontinuous diafiltration (2.0 DV each).

**Figure 2:**
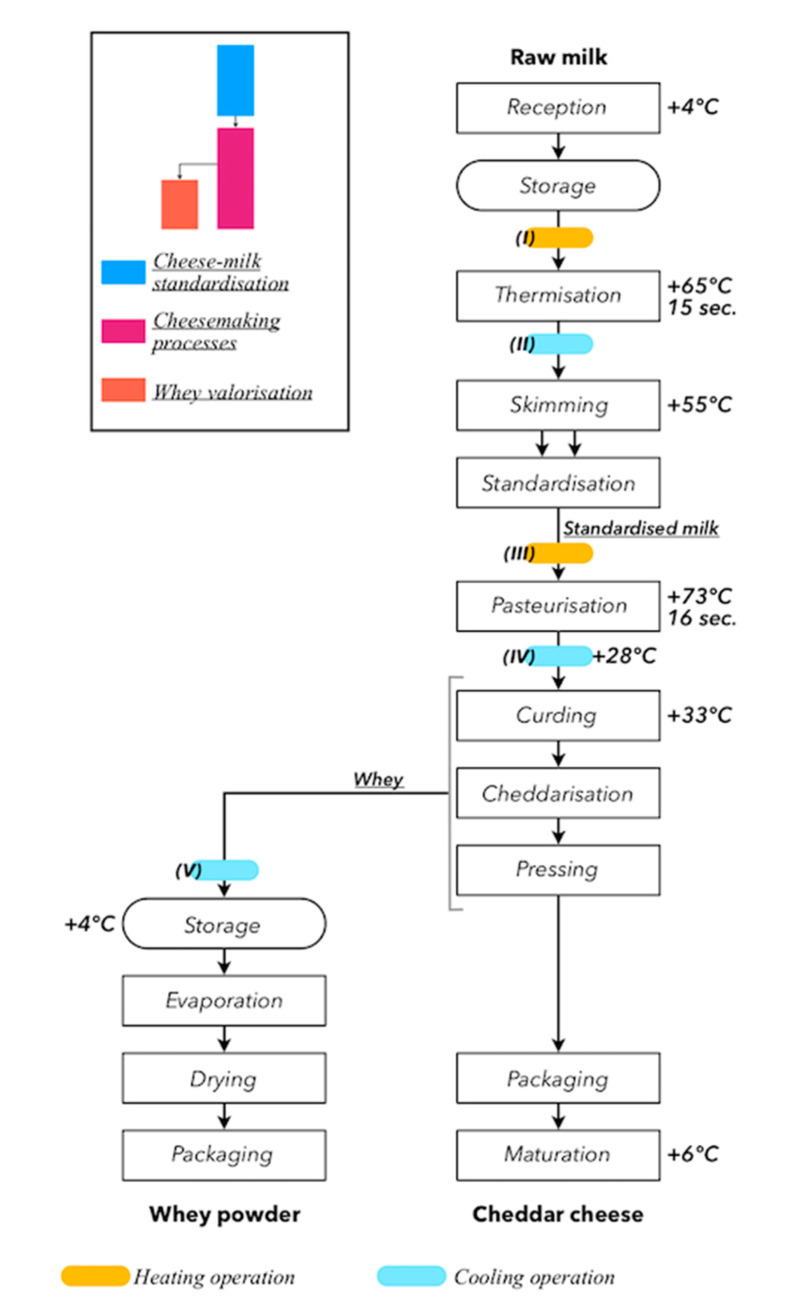
Process flow diagram of scenario C. Based on the information from Tetra Pak [18].

The process flow diagrams of the scenarios are presented in Figure 1 and Figure 2. Three scenarios (A, B, and C) of the dairy process for manufacturing cheddar were compared. After receiving the raw milk, cheese milk was standardized using either UF (scenario A), MF (scenario B), or no membrane separation processes (scenario C). The EE of the three scenarios was assessed from “cradle-to-gate”: from raw milk production to the exit gate of the dairy processing plant where the cheddar is ready for distribution. All dimensions related to the life cycle of raw milk production, transport, and subsequent dairy processing operations were accounted for. The transport, distribution, and consumption of the finished products stages—which are common to the three scenarios—were excluded from this comparative EE assessment. All stages of the life cycle were deemed to take place in the province of Québec (Canada) and the transport distance of raw milk from dairy farms to cheesemaking plants was fixed at 70 km. Each scenario was divided into several production units: a cheese milk standardization unit, a cheesemaking process unit, and a whey valorization unit. A permeate valorization unit was also included in scenarios A and B.

Cheese milk standardization begins with receiving the raw milk at 4 °C and ends with the production of cheese milk with standardized protein and fat contents. The three scenarios shared the same raw milk composition: 3.97% fat (TF), 3.27% true proteins (TP), 4.81% lactose, and 0.75% salts (w·w^−1^) [16]. After standardization, the casein-to-fat ratio (CN/TF) of cheese milk agreed with Ong et al. [10], at 0.67–0.72. In scenarios A and B, the standardization process also allowed for an increase in the cheese milk protein content to 5.93% (w/w), as per Oommen et al. [17].

The cheese milk standardization step of scenario C corresponded to that described by TetraPak [18]: 100% of the raw milk was skimmed prior to standardization (Figure 2). In scenarios A and B, only a portion of the raw milk was skimmed: raw milk, cream, and the retentate generated from the membrane separation processes performed on skim milk (SM) were combined at the standardization stage (Figure 1). All SM generated at the skimming stage was sent to the membrane separation processes for concentration and diafiltration (DF). The membrane separation processes described in Gavazzi-April et al. [19] was used for scenario A, and the membrane separation processes described in Mercier-Bouchard et al. [12] was used for scenario B. In scenario A, SM was concentrated to a volume concentration factor (VCF) of 3.5 then continuous DF with 2.0 diavolumes (DV) was performed—the diavolume is the ratio of the volume of consumed diluent to the volume of treated retentate [20]. These two steps were carried out with UF spiral-wound membranes with molecular weight cut-offs (MWCOs) of 10 kDa. In scenario B, concentration and DF were performed with MF spiral-wound membranes with a pore size of 0.1 µm. SM was concentrated to a VCF of 3.0 and two discontinuous DF of 2.0 DV were performed. In both scenarios, concentration and DF were carried out at 50 °C. For scenario B, the protein rejection coefficients obtained by Mercier-Bouchard et al. [12] were used in the mass balance calculations. In scenario A, and in agreement with El-Gazzar et al. [9], total CN retention was considered.

The three scenarios shared the same constraints: the CN/TF ratio of the standardized cheese milk was kept between 0.67 and 0.72, no overage of cream or SM was generated, and for scenarios A and B, 100% of the retentate was used for cheese milk standardization. This generated only one product at the standardization step: a cheese milk with standard protein and fat contents. However, a byproduct was obtained from cheese milk standardization in scenarios A and B: the permeate from concentration and DF.

The cheesemaking process after the cheese milk standardization step was identical in the three scenarios and corresponded to that described by TetraPak [18] and Fox et al. [16] for the manufacture of cheddar. As detailed by Oommen et al. [17], the following ingredients were used during the cheesemaking process: 0.4 mL of calcium chloride 45% w·w^−1^ per kg of cheese milk, 0.03 mL of annatto colorant per kg of cheese milk, 8.26 g starter culture per kg of protein in cheese milk, 0.11 mL of microbial coagulant enzyme per kg of cheese milk, and 1.90% w·w^−1^ of salt per kg of curd. Cheese blocks of 18 kg were obtained following pressing [21]. They were vacuum-packed in polyethylene (PE) films and stored at 6 °C for 8 months [16].

The whey drained during the cheesemaking process was valorized into whey powder (WP). This step was identical in all scenarios and used the process described by Schuck et al. [22]: the conversion from liquid whey to WP was carried out using a multistage evaporator and a spray drier. Similarly, the permeate generated by scenarios A and B was valorized into permeate powder. WP and permeate powder were packed in paper bags lined with an inner PE bag [23,24] that could hold 25 kg of powder.

The following parameters were the same for the three scenarios: 1500 m^3^ of raw milk were received and each processing plant operated for 20 h, daily. Cleaning and sanitation procedures were carried out during the four remaining hours of the day.

### 2.2. Eco-Efficiency Assessment of the Scenarios

The EE assessments of the scenarios were carried out using the software presented in Benoit et al. [15]. Five eco-efficiency indicators (EEI) were therefore calculated. Four were related to environmental damage and used the Impact2002+ impact analysis method [25]:

- EEI1: Net margin generated per unit of damage to the human health (HH) category,
- EEI2: Net margin generated per unit of damage to the ecosystem quality (EQ) category,
- EEI3: Net margin generated per unit of damage to the climate change (CC) category,
- EEI4: Net margin generated per unit of damage to the non-renewable resources (R) category.

One EEI was related to electricity consumption (EC) at the processing stage [15]:

- EEI5: Net margin generated per unit of electricity consumed at the processing stage.

Since the software was not fitted with constrained optimization features, dimensioning and researching operational settings of all the process elements was required before modeling the three scenarios.

As per the constraints previously described, the dimensioning of the process elements was based on the distribution of raw milk at the cheese milk standardization step—the dimensioning of the process elements was thus different for each scenario. In scenarios A and B, the amounts of cream and retentate generated depended on the share of raw milk sent to the skimming step. That share was calculated using an iterative approach in which it was gradually increased until the protein content of the cheese milk reached 5.93% w·w^−1^. Following the reasoning for the fluid distribution, each process element was dimensioned to cope with the productivity of the processing paths. The plate heat exchangers (nine in scenarios A and B and five in scenario C) were dimensioned and configured to maximize heat regeneration, to minimize the heat transfer surface, pressure losses, and energy use, and to keep the residence time under 10 s. The hydraulic network was dimensioned to maintain the fluid velocity under 3.5 m·s^−1^, as recommended by TetraPak [18]. A natural gas-fired boiler produced the steam used in the production of hot water at 75 °C, and a cooling unit produced water at 2 °C using a water-cooled condenser combined with a cooling tower (as described by TetraPak [18]). As per Benoit et al. [15], the configuration of the cleaning unit was done in accordance with data presented by Yee et al. [26].

### 2.3. Modeling of the Membrane Separation Process

Membrane separation processes were modeled using the method suggested by Cheryan [27] and Global Engineering Alliance (GEA) [28] and described in this section. This method uses filtration flux datasets to dimension a filtration unit to the desired productivity. Filtration datasets were obtained from Gavazzi-April et al. [19] and Mercier-Bouchard et al. [12] (UF and MF, respectively—experiments carried out at the pilot scale in the batch mode). These data showed the evolution of the permeation flux (*J*) as a function of the VCF. They made it possible to determine the relation between (1/*J*) and (*Q_Feed_*/VCF). With *Q_Feed_* the required feed flow rate of the filtration unit (m^3^·h^−1^)—as determined from the fluid distribution calculations—and J the permeation flux (m^3^·h^−1^·m^−2^) from the collected filtration data. When choosing a *VCF* lower than the final *VCF* value, the calculation of (*Q_Feed_*/ VCF) led to the corresponding (1/*J*) value. The (1/*J*) value was either already known from the collected filtration data for that (*Q_Feed_*/VCF) value, or it was calculated by linear interpolation between two existing coordinates. Equations (1), (2), and (3) (which are basic definitions of membrane separation processes concepts, valid at any time during a filtration) then made it possible to calculate the required membrane area of each stage:

*Q_Feed_* = *Q_Ret_* + *Q_Perm_* (1)

(when ignoring density differences between feed, retentate, and permeate)

*Q_Perm._* = *J*·*S* (2)

VCF = *Q_Feed_*/*Q_Ret._* (3)

where *Q_Ret_* and *Q_Perm_* are the retentate and permeate flow rates (m^3^·h^−1^), respectively, and *S* is the membrane area required for this stage (m^2^).

From these equations, the required membrane area for the stage can be calculated using the feed flow rate (*Q_Feed_*) and the coordinates (*Q_Feed_*/VCF; 1/*J*) previously determined:

(4) $$S=\;\left(Q_{Feed}-\frac{Q_{Feed}}{\mathrm{VCF}}\right).\frac{1}{J}$$

The retentate flow rate of the stage was then calculated using Equation (3). By definition, this flow rate is the feed flow rate of the following stage. The same strategy was then implemented from the beginning, adjusting the *VCF* of each stage, until the final *VCF* was reached.

This method was implemented for the dimensioning of the filtration units corresponding to the concentration steps of scenarios A and B (final VCF = 3.5 and 3.0, respectively), and for the DF steps of scenario B (two discontinuous DF, each with 2.0 DV). As suggested by Lutz [29], a linear extrapolation method was used to model the continuous DF (two DV) of scenario A. Although the VCF and DF settings for producing cheese milk intended for cheddar production were non-optimal, this membrane separation processes modeling method can only be implemented using data collected from filtration operations. It is therefore necessary to conform to the filtration data available at the time the process simulations were carried out. It is also worth mentioning that, if available, filtration datasets corresponding to a VCF of 2.8 and a DF of 0.2 DV would have been more suited for cheese milk production. The EC of the membrane separation processes were calculated by summing the consumptions of the electric motor driving the pumps at each stage. These were calculated from the feed flow rate of the stage, the inlet pressure of the membrane modules (in accordance with the pilot scale experiments), and the mechanical efficiencies of the pumps and motors.

### 2.4. Modelling of Cheesemaking, Evaporation, and Drying Processes

The targeted moisture content of the cheeses produced in the three scenarios was 36% w·w^−1^. Cheese yields were calculated using the modified Van Slyke formula (Equation (5)) suggested by Mullan [30] to predict the cheddar manufacturing yield:

*M* = 1703.12 × (0.93 *× %TF* + 0.96 *× %CN*) (5)

where *M* is the mass (kg) of cheddar obtained per ton of standardized cheese milk (moisture content of 36% w·w^−1^), and %*TF* and %*CN* are the percentage (w·w^−1^) of *TF* and *CN*, respectively, in the standardized cheese milk.

Using the same equation for cheddar production from cheese milk standardized with UF retentate (*CN*/*TF* ratio of 0.70, and *CN* concentration of 5.95% w·w^−1^), the yields predicted by Oommen et al. [17] had a confidence interval of 98.6%.

Mass and energy balances for the curding, cheddaring, pressing, and conditioning steps were calculated from the data presented by Sun et al. [21] and TetraPak [31,32].

The energy consumption of the vacuum evaporation step and the drying step of the whey and permeate were predicted using the results obtained by Schuck et al. [22] through process simulation.

### 2.5. Economic Parameters

The economic data related to production costs are presented in Table 1. Most of these data are the mean values of the rates applied in Québec or Canada between the years 2014 and 2018 (for transport, electricity, natural gas, wastewater treatment, building, industrial equipment, and human resources).

To prevent the introduction of biases, the costs of the membrane modules correspond to the prices indicated in Alfa Laval’s catalogue [39] for spiral wound modules with the same specifications as the membranes used in this study. Each module was given a lifetime of 18 months.

The cost of raw milk was based on its composition and the rates of Québec class 3b1: cheddar cheese and related cheeses [33].

A mean cost was selected for fresh water. In Québec, for the majority of residential, commercial or industrial consumers, fresh water is indirectly charged through the municipal taxes without measurement of the volume consumed [37,50]. Taking all sectors into consideration, 95% of Canadian manufacturers use surface or ground water pumping to self-supply [51]. For the three scenarios, a mean cost of $0.53 CAD per m^3^ of water consumed was set, as per MAMROT [37].

Since Canadian market selling prices for the cheese and powders were not available, the selling prices were set from the data available on the American market [52]. The cost of American class III milk (cheese class) was significantly lower at $0.36 CAD·kg^−1^ versus $0.81 CAD·kg^−1^ in Québec. The selling prices were, therefore, increased from the observed American prices until economic profitability was reached for the three scenarios. The obtained price ranges were:

- Cheddar: from $7.50 to $9.50 CAD·kg^−1^;
- WP: from $0.55 to $1.05 CAD·kg^−1^;
- Permeate powder: from $0.45 to $0.85 CAD·kg^−1^.

These prices correspond to a relative increase in the observed American prices (from January to March 2018 [52]) from 1.40 to 1.90. Using the features included in the EE assessment software, it was possible to carry out a sensibility analysis on the profitability of the scenarios depending on the selling prices of the cheddar and powders. The net margin generated per ton of raw milk was therefore evaluated for all possible selling price configurations.

## 3. Results

### 3.1. Mass and Composition of the Dairy Products

The mass and composition of the dairy products obtained for each scenario is presented in Table 2. The masses of cheddar were similar for the three scenarios, with a global yield of about 107 kg of cheddar produced per ton of raw milk processed. Scenario C generated the highest mass of cheddar and scenarios A and B generated 99.7% and 99.1%, respectively, of that mass. For all scenarios, TF, TP, and moisture content of the cheddar were very close and the mean values (37.12%, 25.25%, and 37.43%, respectively) had low standard deviations (0.14%, 0.12%, and 0.10%, respectively). The TP and lactose contents were similar to those presented by O’Brien [53] for cheddar.

Whey presented a dry matter of 6.10%, 4.70%, and 6,84% in scenarios A, B, and C, respectively. It increased to 58.54%, 56.74%, and 61.48% (respectively) after the evaporation step and reached 96.00% for all scenarios after the drying step. Although the masses of cheddar were similar in the three scenarios, the masses of WP were different. The amount of WP generated by scenarios A and B was only 44% and 32%, respectively, of the amount generated by scenario C. The WP composition produced by scenarios A and B was similar (relative variations less than 2%, except for fat), but differed from that of scenario C (Table 2). In scenario C, the WP composition was very similar to the results presented by De Boer [3] for sweet cheese whey. For scenarios A and B, the protein content of the WP was almost twice as high as that of scenario C.

Scenarios A and B generated a total of 36 kg and 45 kg of permeate powder, respectively, for each ton of raw milk processed. Permeates presented a dry matter of 4.25% and 2.94% in scenarios A and B, respectively. It increased to 61.22% and 59.69% (respectively) after the evaporation step and reached 96.00% for both scenarios after the drying step. The permeate powder of scenario B had a TP content 4.3 times higher than that of scenario A. Although the compositions of the generated permeate powder were in accordance with the Codex Alimentarius standards for milk permeate powder [54], it is worth mentioning that, due to its high protein content, the composition of the permeate powder of scenario B is different from that of standard market permeate.

It should be noted that milk losses were not accounted for in these simulations. For each scenario, the solid content of raw milk is thus equal to the sum of the solid contents of the products and coproducts.

### 3.2. Fluid Distribution at the Standardization Step

**Figure 3:**
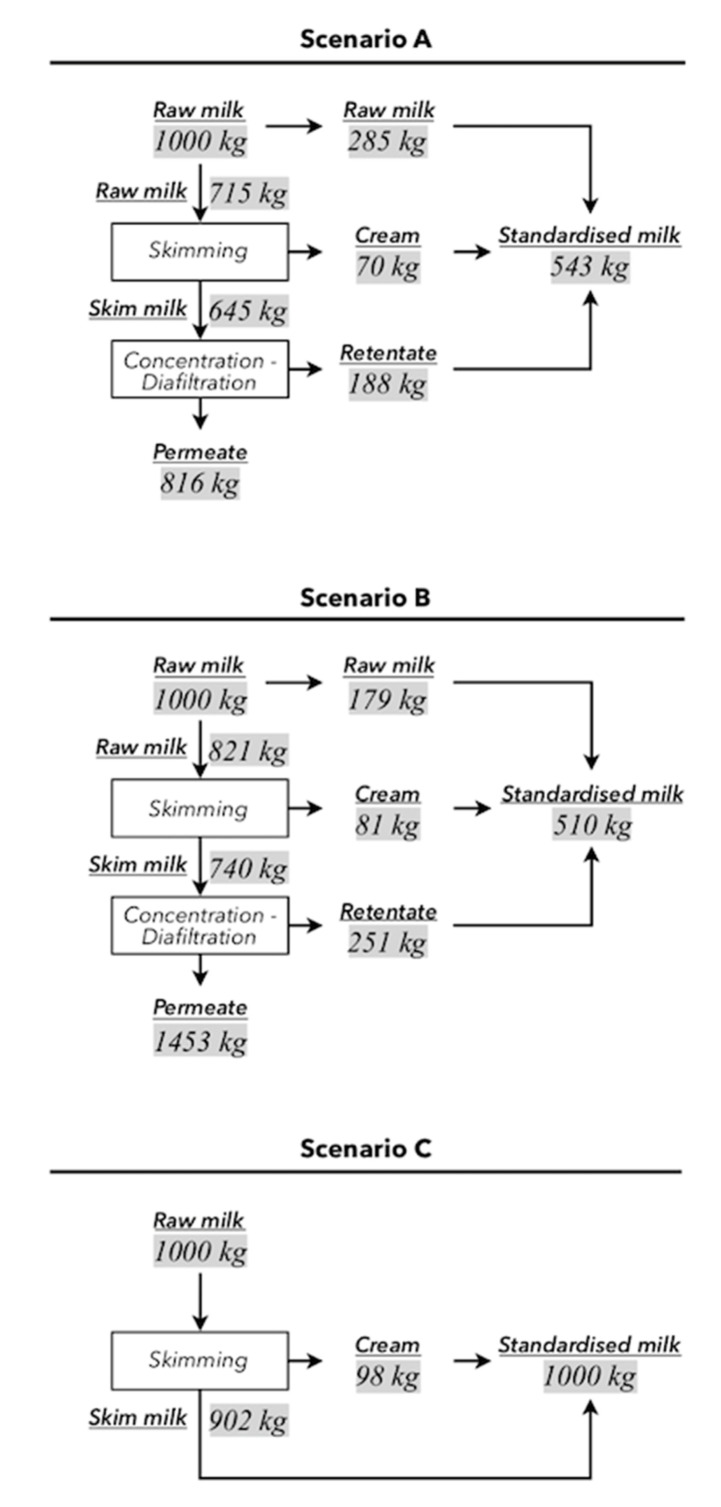
Distribution of the dairy fluids at the standardization step for scenarios A, B, and C.

The fluid mass distribution at the standardization step is presented in Figure 3 and fluid composition is presented in Table 3 and Table 4. Due to the constraints explained above, 0.68 was the only possible CN/TF ratio (Table 3 and Table 4). In scenario C, this resulted in the cheese milk with the same composition as the raw milk, with one ton of raw milk generating one ton of cheese milk. For scenarios A and B, the fluid mass distributions differed: 71.5% of the raw milk was skimmed in scenario A and 82.1% in scenario B.

The SM and cream compositions were the same in the three scenarios. The skimming process produced 9.8% (w·w^−1^) cream (40.0% fat (w·w^−1^)) and 90.2% (w·w^−1^) SM (0.05% fat (w·w^−1^)). In scenario A, 29% of the SM mass was converted into retentate and 34% was converted in scenario B. The TP content (w·w^−1^) of the retentate was higher in scenario A (11.42%) than in scenario B (9.08%). However, the proportion of CN (related to the total mass of TP) was higher in the scenario B (89%) retentate than in the scenario A (84%) retentate. In both scenarios, concentration and DF steps produced a higher CN/TP ratio in the retentate than in the SM.

For each ton of raw milk received, 816 kg and 1453 kg of permeate were produced in scenarios A and B, respectively. In scenario B, the TP content of the permeate (w·w^−1^) was three times higher than in the scenario A permeate, and the TS content was one third lower (Table 4). The mass of permeate was higher in scenario B and each ton of permeate generated 44% more permeate powder than scenario A (31 kg and 44 kg, respectively).

Cheese milk was composed of raw milk, cream, and retentate. In scenario C, one ton of raw milk generated one ton of cheese milk. In scenarios A and B, one ton of raw milk generated 543 kg and 510 kg of cheese milk, respectively. Crossing these results with those of Table 2 makes it appear that, since all scenarios generated almost the same mass of cheddar, the vat cheese yields were thus nearly two times higher in scenarios A and B than in scenario C. The cheese milk from scenario B met the imposed constraints: the TP content was 5.93% w·w^−1^ (versus 3.27% in scenario C), and the CN/TF ratios observed were between 0.67 and 0.72. Since the retentates had different compositions, the cheese milk compositions were also different. While the cheese milk obtained in scenario A was 52% raw milk, 13% cream, and 35% retentate (w·w^−1^), the scenario B cheese milk was 35% raw milk, 16% cream, and 49% retentate (w·w^−1^). The sources of CN also differed between these two scenarios: 29%, 4%, and 67% (w·w^−1^) of the CN originated from raw milk, cream, and retentate, respectively, in scenario A, compared to 18%, 5%, and 77% in scenario B. Even if the masses of cheese milk in scenarios A and B were nearly half of that of scenario C, the masses of cheddar obtained were almost identical in the three scenarios (Table 2).

### 3.3. Dimensioning of the Filtration Units

**Figure 4:**
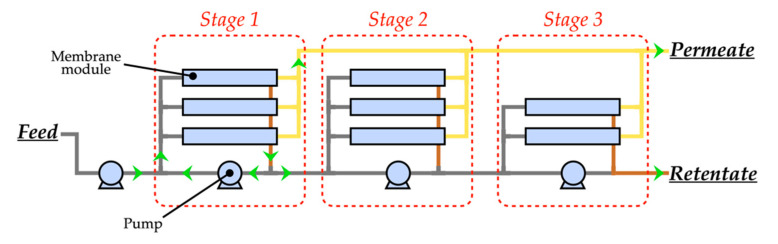
Process flow diagram of the multistage filtration units of scenarios A and B.

The filtration units were dimensioned according to the method presented in Section 2.3. The results are presented in Table 5. Concentration steps were carried out in four stages for both scenarios, and the targeted VCF were reached. Three stages were used for both the continuous DF of scenario A and the two discontinuous DF of scenario B. A process flow diagram of the filtration units is presented on Figure 4. As previously mentioned, the amount of membrane surface required was predicted from the feed flow rate values, the VCF, and the experimental permeation fluxes. Although the feed flow rate of the filtration unit was lower in scenario A (Figure 3), the membrane surface required was higher in this scenario: 6221 m^2^, compared to 3071 m^2^ in scenario B (Table 5).

In scenario A the stage flow rates were lower by 30%, on average, than in scenario B. The operating parameters were different in both scenarios, and the inlet pressure was 39% lower for MF (scenario B). However, the additional DF step in scenario B drove up the total power requirement of the filtration unit to 42.6 kW, which exceeded that of scenario A (34.9 kW; Table 5). It is worth noting that the filtration units were operated daily for 20 h in both scenarios.

### 3.4. Eco-Efficiency Indicators

The EEI and potential environmental impacts values are shown in Table 6. The values correspond to the costs presented in Table 1 and the following selling prices: $8.50 CAD·kg^−1^ of cheddar, $0.80 CAD·kg^−1^ of WP, and $0.60 CAD·kg^−1^ of permeate powder.

As shown in Table 6, scenario C had the highest EEI values and scenario B, the lowest, irrespective of the environmental damage category. The EEI values of scenario A were 8–16% higher than those of scenario B, whereas the scenario C EEI values were 18–30% higher. The highest and the lowest differences were observed for EEI5 and EEI2, respectively. In this economic context, membrane separation processes contributed negatively to the EE of raw milk valuation.

The potential environmental impacts were less important in scenario C, regardless of the damage category. However, the differences with the other scenarios were low. The differences in EEI values between scenarios A and C were less than 1% for the HH, EQ, and CC damage categories, and just 2.6% for the R category. Similarly, the differences between scenarios B and C were less than 1.2% for the first three damage categories and just 3.3% for the R category.

The EC values were the same order of magnitude for the three scenarios and followed the same patterns as the four other EEI. Scenario C had the lowest value, followed by scenarios A (+2.4%) and B (+10.5%). Unlike the EEI related to damage categories, the EC only considers the processing stage of the raw milk life cycle [15]. Therefore, the observed differences were directly related to the modeling parameters of the scenarios.

### 3.5. Contributions to Potential Environmental Impacts

The mean contributions of the potential environmental impacts to the raw milk life cycle are presented in Table 7.

Regardless of the scenario and the damage category, raw milk production was the main contributor to the potential environmental impacts. On average, it was responsible for more than 91% of the damage in the HH, EQ, and CC categories (standard deviation less than 1%), and 75% (±1%) of the damage in the R category. The transport stage contributed equally to the potential environmental impacts of the three scenarios. Its maximum contribution was 2% of the damage in the R category. The contribution of the processing stage to the potential environmental impacts ranged from 1% (standard deviation less than 1%) to 23% (±1%), for damage in the QE and R categories, respectively.

Even if the contributions of raw milk production and transport to the potential environmental impacts were different for each scenario, the potential environmental impacts of these life-cycle stages were equal for the three scenarios. Indeed, the volume of raw milk received at the processing plants and the transport needs were identical in each scenario. This made it possible to narrow the comparison to the processing stage of the raw milk life cycle (excluding raw milk production and transport) without introducing bias.

Table 8 presents the potential environmental impacts of the processing stage and the contributions of the reference flows to the potential environmental impacts. For the four damage categories, scenario B produced the highest impact and scenario C, the lowest. The widest gap between these two scenarios was observed for the damage in the EQ category, with a difference of 25%. Overall, the results of the three scenarios showed similar trends. Natural gas was an important contributor to all the damage categories. Its contributions were greater than 86% and 82% in the CC and R damage categories, respectively, which explains why the contribution of the processing stage to the raw milk life cycle damage was higher for this last damage category (Table 7). The contribution of electricity was also important. It was the first (second for scenario A) and third highest contributor to the HH and the EQ damage categories, respectively. Finally, wastewater treatment was the main contributor to the damage in the EQ category. Compared to scenario C, wastewater treatment contributed 11% and 30% more in scenarios A and B, respectively.

In addition to the contributions to the potential environmental impacts, the software provided the contributions of each processing step to the total EC. These results are presented in Table 9. The whey and permeate valorization steps were the most electricity-demanding processes. The contributions of these processes totaled 83%, 78%, and 79% for the scenarios A, B, and C, respectively. For the scenarios A and B, the membrane separation processes were the second contributors, followed by the skimming step. The second and third contributor in scenario C were the skimming and curding step.

### 3.6. Economic Viability

Table 10 shows the results of the economic viability assessment for the three scenarios. Twenty-seven economic conditions, based only on the selling prices of the products, were assessed. Scenario C was the most profitable in two thirds of these economic conditions, and scenario A was the most profitable in the remaining third. With a cheddar price of $7.50 CAD·kg^−1^, scenario C was the only profitable scenario, but only when the price of the WP was at least $1.05 CAD·kg^−1^ (with a profitability threshold at $0.95 CAD·kg^−1^). With a cheddar price of $8.50 CAD·kg^−1^ and up, a positive net margin was observed for all scenarios. At a WP selling price of $1.05 CAD·kg^−1^, scenario C was always the most profitable, regardless of the cheddar and permeate powder prices considered. 

**Figure 5:**
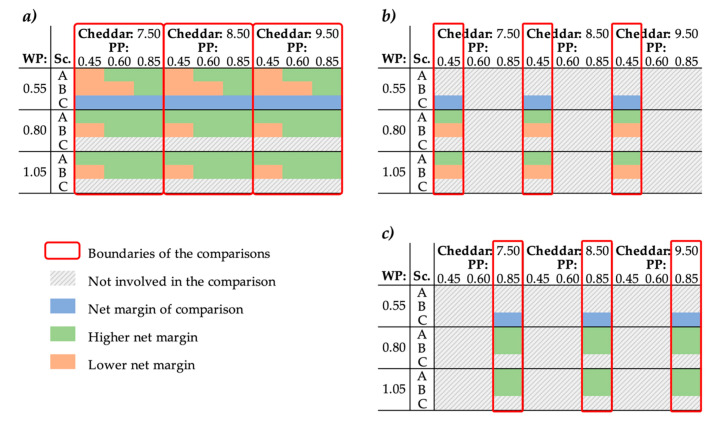
Comparisons of the net margin generated per ton of raw milk received as a function of the selling prices ($CAD·kg^−1^) of the product and coproducts from scenarios A, B, and C for whey powder selling prices lower in scenario C than in scenarios A and B. (**a**) All permeate powder (PP) prices. (**b**) Lowest PP price. (**c**) Highest PP price. WP: Whey Powder, Sc.: Scenario, PP: Permeate Powder.

Since the WP had different compositions in each scenario, they could also have different selling prices. Figure 5 presents the net margin comparisons carried out for whey powder selling prices lower in scenario C than in scenarios A and B. When compared with the net margin generated by scenario C at the same cheddar selling price but kept at the lowest WP selling price (Figure 5a), scenarios A and B were more profitable in 89% and 56% (respectively) of the 27 suggested economic conditions. At the same cheddar selling price, the lowest permeate powder selling price, and higher WP selling prices than scenario C (Figure 5b), scenario A was 1–10% more profitable, and scenario B generated lower net margins than scenario C. In the same conditions but at the highest permeate powder selling price (Figure 5c), scenarios A and B were always more profitable than scenario C, with net margins higher by 8–28% for scenario A, and 6–20% for scenario B. It is important to mention that for each economic condition, the most profitable scenario was also the most eco-efficient. Scenario B thus produced the lowest EE for each economic condition assessed.

## 4. Discussion

### 4.1. Process Yields

Given the masses of cheddar generated in the three scenarios (Table 2), it appeared that the introduction of membrane separation processes for cheese milk standardization did not improve the global yield (mass of cheddar generated per ton of raw milk). Heino et al. [55] came to the same conclusion for Edam cheese. However, although the cheese milk masses in scenarios A and B were almost half the mass in scenario C, the fat and protein enrichment of the scenario A and B cheese milks produced the same mass of cheddar as scenario C. As observed by Oommen et al. [17], the vat cheese yields were thus nearly two times higher in scenarios A and B.

The slightly lower global yield of scenario B (Table 2) was likely due to the loss of CN during the membrane separation processes step. In this scenario, the CN/TF ratio of the cheese milk is one hundredth of a point lower (Table 4). Compared to UF (10 kDa), protein transmission into permeate was greater in MF (0.1 µm). For MF, the global rejection coefficients were 0.99 and 0.95 for CN and serum proteins, respectively, whereas they were 1.00 and 0.99 for UF [12,19].

Even if limited, the transmission of proteins during the membrane separation processes steps explains most of the differences observed between the dairy fluids and products generated in the three scenarios. The higher CN/TP ratio of the retentate in scenario B (Table 4) is a direct consequence of this transmission. Higher whey protein transmission increased the CN/TP ratio. The purification carried out during the DF steps reduced the lactose and mineral contents of the retentates and increased that of the permeates (Table 4). Consequently, the cheese milk produced by scenarios A and B had lower lactose and mineral content than that of scenario C (Table 4). The smaller masses of whey and their lower lactose and mineral content in scenarios A and B is thus due to the reduced content of lactose and minerals in cheese milk and the higher vat yields of these scenarios (Table 2). In return, the WP was enriched in serum proteins (Table 2), which explains why the TP content of the WPs were higher than reported by De Boer [3]. Similarly, the lower rejection coefficients combined with the greater number of DV in scenario B explained the higher TP content of its permeate powder (Table 2). Although the retentate of scenario B was purified further, more protein was lost.

It is however important to stress that because VCF and DF settings were non-optimal for cheddar production, the cheese milk generated might not allow for the production of reasonable quality cheddar. Unfortunately, the quality of the products could not be predicted from the results of the simulations.

Since the filtration datasets used for the dimensioning of the filtration units were obtained from different operating conditions (UF with a VCF of 3.5 and one DF of 2.0 DV versus MF with a VCF of 3.0 and two DF of 2.0 DV), the membrane surface required for scenario A is more than twice the membrane surface of scenario B (Table 5). The use of filtration datasets generated in operating conditions that would allow for higher permeation flows in UF or lower permeation flows in MF would most likely lead to a smaller difference in the membrane surfaces of scenarios A and B.

Finally, it would be interesting to take the processing losses into account since they could have significant impacts on the process yields and, by extension, on the values of the EEIs.

### 4.2. Effects of the Differentiated Distribution of the Fluids

As presented in Section 3.2, due to the constraints imposed on the three scenarios, the dairy fluids (raw milk, SM, cream, and retentate) are distributed differently between the process operations of the standardization step in each scenario. In scenarios A and B, three paths led the dairy fluids from the raw milk reception to the standardization unit (Figure 3). The consumption (resources and energy) and generation (effluents and emissions) associated with each path were expected to depend on the complexity of the operations and the distribution of the fluids to each path. Therefore, since 82% of the raw milk was directed to the two most complex paths in scenario B (compared to 71% in scenario A), it was not surprising that the EC of the skimming and standardization steps were higher in scenario B.

The mass of the permeate in scenario B was increased not only by the differentiated fluid distribution but also by a higher number of DV than in scenario A. As a result, the EC of the membrane separation processes and the cooling unit were higher in scenario B than in scenario A (Table 9). Indeed, the amount of permeate to be cooled in scenario B was nearly double that of scenario A (exchanger V, Figure 1; Figure 3).

Scenarios A and B are alternative choices of the same process (Figure 1), and process modeling only differed for two operations out of 22: the concentration and DF steps (different membrane types and operating modes). However, in terms of EC, the differences in the membrane separation processes consumptions of scenarios A and B explained only 38% of the total difference in EC, leaving 62% of the difference attributable to the indirect effects of the differences in membrane separation processes modeling (Table 9). Process simulation is advantageous for such a comparison since it is then impossible to miss the indirect, and potentially important, effects induced by different operational settings. By default, process simulation accounts for direct and indirect effects of each modeling setting and allows for easy identification of these effects. Carrying out such a study without using process simulation would require one to specifically research these effects or run the risk of disregarding them.

### 4.3. Compared Eco-Efficiencies

In terms of EE, scenario C is doubly advantageous. It had the lowest potential environmental impacts and was the most profitable in most economic contexts. Scenario C was therefore the most eco-efficient scenario in most economic contexts. In the economic contexts in which scenario C was not the most eco-efficient, scenario A seemed to be the most eco-efficient, but scenario C had the lowest potential environmental impacts for all damage categories.

Since raw milk production contributed to over 70% of the potential environmental impacts in all scenarios (Table 7), any scenario that might increase the global cheese yield would probably increase the EE of the process. However, the results showed that the introduction of membrane separation processes at the cheese milk standardization step of cheddar production did not increase the global cheese yields.

Reducing natural gas consumption would benefit all scenarios and reduce the potential environmental impacts of the processing stage (Table 8). Natural gas contributes so much to the CC and R damage categories (Table 8) because it is a fossil fuel for which extraction, treatment, and combustion emit greenhouse gases, and its extraction contributes to non-renewable energy use [56]. Similarly, since the packaging materials were made from petroleum-based materials, they were the second highest contributor to the CC and R damage categories. Breaking down Schuck et al.’s [22] models showed that the production of WP and permeate powder were responsible for 67%, 66%, and 72% of the total natural gas use in scenarios A, B, and C, respectively. As suggested by the United Nations Environment Programme (UNEP) [57] and GEA [58], one way to reduce the consumption of natural gas in the production of dairy powders would be to add a reverse-osmosis (RO) step prior to evaporation and drying. Introducing nanofiltration (NF) or RO operations followed by UF could not only help reduce natural gas consumption, but also generate high value byproducts such as whey protein isolates and concentrates, and lactose powder [8]. These additions could reduce natural gas consumption and generate smaller amounts of coproducts [3], which sell at prices at least four times higher than whey powder [52], potentially improving the EE of the scenarios. It is also worth mentioning that since no crystallization step was applied to the permeates, the energy consumption (in terms of electricity and natural gas) could have been underestimated in the three scenarios. The powder compositions could also be affected by the introduction of a crystallization step. It would thus be worth investigating that addition to the modeled processes.

Wastewater treatment was the main contributor to the potential environmental impacts of the processing stage in the EQ category (Table 8). Results showed that wastewater originated mainly from the cleaning and sanitizing operations, and from the evaporation steps of WP and permeate powder productions. Once again, the introduction of additional membrane separation processes might improve the EE of the scenarios. Membrane separation processes such as UF, NF, or RO could be used to regenerate the cleaning and sanitizing solutions [59], potentially reducing wastewater volumes and the need for fresh detergents. Additionally, vapor condensates could be recycled [58,60] using RO operations to limit the volume of effluent generated [57]. Although such solutions appear promising, complete EE assessments would be required to confirm these hypotheses. It would indeed be difficult to estimate the effects of the installation and operations of one or several RO units capable of handling such volumes on the operation costs and environmental impacts of the whole process without a proper assessment.

### 4.4. Realism of Modeling of the Filtration Units

The modeling method for this study used pilot-scale datasets to model industrial-scale filtration units. However, several incompatibilities between these two scales might work against the realism of the simulation results.

The performance of membrane separation processes depends on many parameters. The membrane properties, filtration temperature, fluid properties, tangential flow velocity, and the trans-membrane pressure (TMP) have significant effects on permeate and retentate composition, permeation flux, the fouling phenomenon, and pumping power requirements [27,61,62]. In multi-stage industrial filtration systems, operating conditions might differ at every point in the system. For example, when placing filtration modules in series, the tangential velocity and TMP decrease along the membrane housing [28]. Each membrane is therefore operated under different parameters. Additionally, since the pumps are a centrifugal type, at a constant rotation speed, the flow rate is diminished while fouling increases, resulting in a reduced tangential flow velocity. Finally, recirculation of a portion of the retentate in each stage directly impacts the tangential flow velocity, and indirectly affects the flow rate of the feed pump, and thus the TMP of each stage. Yet, in this study’s modeling method, the datasets were generated at a fixed TMP and with an unknown tangential flow velocity—moreover, the filtration unit used by Gavazzi-April et al. [19] was not fitted with a recirculation loop. It is therefore possible that the results from the simulations of the membrane separation processes were not totally realistic.

From a technical point of view, filtration systems are complex hydraulic networks. A modeling and simulation method that includes a solving method for the hydraulic network analysis—such as the solving method presented by Robert [63] for water and sewer networks—would make it possible to realistically calculate the flow rates and pressures at any point in a filtration system for the entire duration of the operation. Furthermore, using complete filtration datasets generated for a whole range of operating conditions, as presented by D’Souza et al. [64], would offset the biases noted above, and facilitate realistic modeling, simulation, and analysis of industrial-scale filtration systems. A modeling and simulation method like the one described here would make it possible to predict the material and energy balance of the filtration units of any scale and configuration (including feed and bleed and multistage configurations) with more realism than the method currently available.

## 5. Conclusions

In the present case study, the assessment and comparison of the EE of three scenarios of cheddar production showed that the introduction of membrane separation processes at the standardization step with the chosen parameters (UF with a VCF of 3.5 and one DF of 2.0 DV or MF with a VCF of 3.0 and two DF of 2.0 DV) negatively impacted the EE of the process in the Québec context. The scenario devoid of any membrane separation processes was the most eco-efficient in most economic contexts and it had the lowest potential environmental impacts for all damage categories. It seems, however, that introducing membrane separation processes at the valorization steps of whey and permeate might improve the EE of the process. These additional scenarios should be modeled and assessed to draw a conclusion on the contribution of membrane separation processes to the global EE of dairy processes. While this study clearly shows the benefit of process simulation for assessing complex scenarios, its results should be backed up in the future with either industrial data or more realistic membrane models. To that end, generating filtration datasets for a range of operating conditions is needed. An improved modeling method for the membrane separation processes would use these datasets to create realistic simulations of industrial-scale filtration systems from pilot-scale datasets.

## Figures and Tables

**Table 1:** List of the costs considered in scenarios A, B, and C.

Descriptions	Costs	References
Raw milk	$0.81 CAD·kg^−1^	[33]
Milk transport	$23.23 CAD·t^−1^	[34]
Electricity	$0.038 CAD·kWh^−1^	[35]
Natural gas	$0.0035 CAD·MJ^−1^	[36]
Fresh water	$0.53 CAD·m^−3^	[37]
Acid detergents	$3.72 CAD·kg^−1^	[38]
Alkaline detergents	$3.56 CAD·kg^−1^	[38]
Wastewater treatment	$0.45 CAD·m^−3^	[37]
UF membrane (10 kDa)	$82.36 CAD·m^−2^	[39]
MF membrane 0.1 µm	$94.64 CAD·m^−2^	[39]
Starter culture	$18.80 CAD·kg^−1^	[40,41]
Calcium chloride 45% (*w*/*w*)	$4.75 CAD·kg^−1^	[42]
Coagulant enzyme	$21.13 CAD·kg^−1^	[43,44]
Annatto colorant	$2.77 CAD·kg^−1^	[45]
Salt	$0.80 CAD·kg^−1^	[46]
Packaging material	$2.00 CAD·kg^−1^	— ^1^
Building (concrete)	$0.0068 CAD·m^−3^	[47]
Equipment (steel)	$0.0055 CAD·kg^−1^	[48]
Manpower	$14.56 CAD·h^−1^	[49]

^1^ The price of packaging materials was arbitrarily fixed.

**Table 2:** Mass and composition of the raw milk, product, and coproducts generated in scenarios A, B, and C per ton of raw milk processed.

Mass and Composition	Raw Milk	Sc. A			Sc. B			Sc. C	
		Cheddar Cheese	Whey Powder	Permeate Powder	Cheddar Cheese	Whey Powder	Permeate Powder	Cheddar Cheese	Whey Powder
Mass (kg)	1000	107	28	36	106	20	45	107	63
Composition (% *w*/*w*)									
Fat	3.97	37.03	0.72	—	37.27	0.80	—	37.05	0.25
Protein ^1^	3.27	25.12	19.55	1.27	24.90	19.21	5.51	25.13	9.25
Lactose	4.81	0.17	66.23	81.97	0.13	66.49	78.30	0.18	75.26
Salts	0.75	0.24	9.50	12.76	0.18	9.49	12.19	0.32	11.24
Water	87.20	37.44	4.00	4.00	37.51	4.00	4.00	37.32	4.00

^1^ True Protein.

**Table 3:** Composition of the raw milk, skim milk, and cream at the standardization step for scenarios A, B, and C.

	Scenario A, B, and C		
	Raw Milk ^1^	Skim Milk	Cream
Composition (% *w*/*w*)			
Fat	3.97	0.05	40.00
Proteins ^2^	3.27	3.40	2.04
Lactose	4.81	5.01	3.01
Salts	0.75	0.78	0.47
Water	87.20	90.76	54.48
Ratios			
CN/TP	0.82	0.82	0.82
CN/TF	0.68	55.82	0.04

^1^ From Fox et al. [16], ^2^ True Protein; Std milk: Standardized milk, CN/TP: Casein to true protein ratio, CN/TF: Casein to total milk fat ratio.

**Table 4:** Composition of the retentates, permeates, and standardized milks at the standardization step for scenarios A, B, and C.

	Scenario A			Scenario B			Scenario C
	Retentate	Permeate	Std Milk	Retentate	Permeate	Std Milk	Std Milk
Composition (% *w*/*w*)							
Fat	0.17	—	7.31	0.15	—	7.78	3.97
Proteins ^1^	11.42	0.06	5.93	9.08	0.17	5.93	3.27
Lactose	1.45	3.62	3.41	0.90	2.40	2.60	4.81
Salts	0.23	0.56	0.53	0.14	0.37	0.41	0.75
Water	86.73	95.76	82.82	89.73	97.06	83.28	87.20
Ratios							
CN/TP	0.84	0.00	0.83	0.89	0.15	0.87	0.82
CN/TF	55.76	—	0.68	54.76	—	0.67	0.68

^1^ True Protein; Std milk: Standardized milk, CN/TP: Casein to true protein ratio, CN/TF: Casein to total milk fat ratio.

**Table 5:** Dimensioning of the filtration systems for scenarios A and B.

Scenario	Step	Stage	*S*	*J*	sVCF	gVCF	P
			m^2^	L·h^−1^·m^−2^	—	—	kW
A	Concentration VCF = 3.5X	1	987	16.5	1.5	1.5	8.9
		2	671	13.9	1.4	2.1	5.9
		3	486	11.1	1.3	2.7	4.2
		4	474	8.8	1.3	3.5	3.3
	Cont. DF 2.0 DV	1	1376	6.8	1.0	1.0	4.2
		2	1190	7.8	1.0	1.0	4.2
		3	1037	9.0	1.0	1.0	4.2
B	Concentration VCF = 3.0X	1	564	33.2	1.5	1.5	6.2
		2	371	28.8	1.4	2.1	4.2
		3	213	25.1	1.2	2.6	3.0
		4	118	22.9	1.1	3.0	2.4
	Discont. DF #1 2.0 DV	1	435	43.1	1.5	1.5	6.2
		2	310	37.5	1.4	2.2	4.2
		3	224	31.8	1.4	3.0	3.0
	Discont. DF #2 2.0 DV	1	372	50.4	1.5	1.5	6.2
		2	264	44.0	1.4	2.2	4.2
		3	200	35.5	1.4	3.0	3.0

*S*: Membrane surface, *J*: Permeation flux, sVCF: Stage volume concentration factor, gVCF: Global volume concentration factor, P: Power requirement, DV: Diavolume, Cont. DF: Continuous Diafiltration, Discont. DF: Discontinuous diafiltration.

**Table 6:** Values of the eco-efficiency indicators (EEIs), the potential environmental damages, and the electricity consumptions (ECs) per ton of raw milk received for scenarios A, B, and C.

Indicators	Scenarios			Units
	A	B	C	
Eco-efficiency				
EEI1	4.95 × 10^4^	4.57 × 10^4^	5.44 × 10^4^	$CAD/DALY
EEI2	0.058	0.053	0.063	$CAD/PDF m^2^ year
EEI3	0.084	0.077	0.092	$CAD/kg CO_2_ eq.
EEI4	0.011	0.010	0.012	$CAD/MJ
EEI5	3.76	3.23	4.21	$CAD/kWh
Environmental damage				
Human health (HH)	1.81 × 10^−3^	1.82 × 10^−3^	1.80 × 10^−3^	DALY
Ecosystem quality (EQ)	1558	1562	1555	PDF·m^2^·year
Climate change (CC)	1073	1076	1063	kg CO_2_ eq.
Resources (R)	8436	8495	8223	MJ
Electricity consumption (EC)	23.88	25.77	23.32	kWh

DALY: Disability-Adjusted Life Year; PDF: Potentially Disappeared Fraction of species; CO_2_ eq.: CO_2_ equivalent.

**Table 7:** Mean contributions of the raw milk life cycle to each damage category for scenarios A, B, and C ^1^.

Life Cycle Stages	Contributions to the Damage Categories (%)			
	HH	EQ	CC	R
Production	92	98	91	75 ± 1
Transport	1	1	1	2
Processing	7	1	8	23 ± 1

^1^ Standard deviations not presented were lower than 1%; HH: Human health, EQ: Ecosystem quality, CC: Climate change, R: Resources.

**Table 8:** Contributions of the reference flows to the damage categories of the processing stage for scenarios A, B, and C.

Reference Flows	Contributions to the Damage Categories (%)											
	HH			EQ			CC			R		
	A	B	C	A	B	C	A	B	C	A	B	C
Electricity	41.4	41.4	43.7	12.6	11.6	13.9	1.1	1.1	1.2	1.4	1.5	1.5
Natural gas	42.0	40.2	39.2	34.0	29.9	33.2	88.4	88.4	86.5	84.5	84.8	82.2
Fresh water	1.5	2.4	1.0	0.7	1.0	0.5	-	-	-	-	-	-
Acid detergent	0.6	0.5	0.8	2.9	2.5	4.2	-	-	-	-	-	-
Alkaline detergent	2.2	2.0	3.0	1.3	1.1	1.9	-	-	-	-	-	-
Wastewater treatment	5.9	7.5	5.1	40.0	46.9	36.2	-	-	-	-	-	-
Packaging materials	5.1	4.7	5.7	5.4	4.6	6.2	7.5	7.3	8.5	11.6	11.3	13.1
Others	1.3	1.2	1.6	3.0	2.5	4.0	3.0	3.2	3.8	2.4	2.5	3.2
	(10^−4^ DALY)			(PDF m^2^ year)			(kg CO_2_ eq.)			(MJ)		
Damage Value *:	1.23	1.33	1.14	23.0	27.1	20.4	83.4	86.2	73.7	1958	2018	1745

* Per ton of raw milk; HH: Human health, EQ: Ecosystem quality, CC: Climate change, R: Resources, DALY: Disability-Adjusted Life Year, PDF: Potentially Disappeared Fraction of species, CO_2_ eq.: CO_2_ equivalent, -: results included in the “Others” category.

**Table 9:** Contribution of operating steps to the electricity consumption (EC) of the processing stage for scenarios A, B, and C.

Processing Step	Contributions to the EC (%)		
	Scenario A	Scenario B	Scenario C
Skimming	3.6%	4.2%	4.9%
Thermization	0.4%	0.8%	1.4%
Concentration—Diafiltrations	3.9%	6.4%	0.0%
Pasteurization	0.5%	0.6%	3.0%
Refrigeration	1.9%	3.2%	2.5%
Curding	1.2%	1.1%	3.2%
Cheddaring	1.9%	1.8%	2.0%
Pressing—Packaging	2.6%	2.4%	2.6%
Whey processing	33.6%	22.2%	79.1%
Permeate processing	49.3%	56.3%	0.0%
Cleaning and disinfection	0.9%	0.8%	1.1%
Other	0.3%	0.3%	0.2%
EC ^1^ (kWh)	23.88	25.77	23.32

^1^ EC calculated per ton of raw milk.

**Table 10:** Net margin generated per ton of raw milk received as a function of the selling prices ($CAD·kg^−1^) of the product and coproducts from scenarios A, B, and C.

Whey Powder:	Scenarios	Cheddar: 7.50			Cheddar: 8.50			Cheddar: 9.50		
		Permeate Powder:			Permeate Powder:			Permeate Powder:		
		0.45	0.60	0.85	0.45	0.60	0.85	0.45	0.60	0.85
**0.55**	A	−28.86	**−23.44**	**−14.42**	77.83	**83.24**	**92.26**	184.51	**189.93**	**198.95**
	B	−34.12	−27.45	−16.32	71.96	78.64	89.76	178.04	184.72	195.84
	C	**−24.28**	−24.28	−24.28	**82.74**	82.74	82.74	**189.77**	189.77	189.77
0.80	A	−22.34	−16.92	**−7.90**	84.35	89.76	**98.78**	191.03	196.45	**205.47**
	B	−29.50	−22.82	−11.69	76.58	83.26	94.39	182.66	189.34	200.47
	C	**−8.85**	**−8.85**	−8.85	**98.17**	**98.17**	98.17	**205.20**	**205.20**	205.20
1.05	A	−15.42	−10.01	−0.99	91.26	96.67	105.69	197.94	203.36	212.38
	B	−24.60	−17.92	−6.79	81.48	88.16	99.29	187.56	194.24	205.37
	C	**7.01**	**7.01**	**7.01**	**114.03**	**114.03**	**114.03**	**221.06**	**221.06**	**221.06**

Bold values represent the most profitable (or the least detrimental) scenario for each economic condition.
